# Evaluation of tumor immunity after administration of conditionally replicative adenoviral vector in canine osteosarcoma patients

**DOI:** 10.1016/j.heliyon.2021.e06210

**Published:** 2021-02-10

**Authors:** Payal Agarwal, Elizabeth A. Gammon, Maninder Sandey, Stephanie S. Lindley, Jey W. Koehler, Brad M. Matz, Annette N. Smith, Elena A. Kashentseva, Igor P. Dmitriev, David T. Curiel, Bruce F. Smith

**Affiliations:** a Scott-Ritchey Research Center, College of Veterinary Medicine, Auburn University, USA; b Department of Pathobiology, College of Veterinary Medicine, Auburn University, USA; c Department of Radiation Oncology, Washington University School of Medicine in St. Louis, USA; d Department of Clinical Sciences, College of Veterinary Medicine, Auburn University, USA

**Keywords:** Dog, Cancer, Osteosarcoma, Tumor immunology, Canine adenovirus type 2

## Abstract

Osteosarcoma is one among the most common neoplasms in dogs. Current treatments show limited efficacy and fail to prevent metastasis. Conditionally replicative adenoviruses (CRAd) replicate exclusively in targeted tumor cells and release new virus particles to infect additional cells. We proposed that OC-CAVE1 (CAV2 with the E1A promoter replaced with the osteocalcin promotor) may also enhance existing immunity against tumors by overcoming immune tolerance via exposure of new epitopes and cytokine signaling. Eleven client-owned dogs with spontaneously occurring osteosarcomas were enrolled in a pilot study. All dogs were injected with OC-CAVE1 following amputation of the affected limb or limb-sparing surgery. Dogs were monitored for viremia and viral shedding. There was minimal virus shedding in urine and feces by the 6th day and no virus was present in blood after 4 weeks. CAV-2 antibody-titers increased in all of the patients, post-CRAd injection. Immunological assays were performed to monitor 1) humoral response against tumors, 2) levels of circulatory CD11c + cells, 3) levels of regulatory T cells, and 4) cytotoxic activity of tumor specific T cells against autologous tumor cells between pre-CRAd administration and 4 weeks post-CRAd administration samples. Administration of the CRAd OC-CAVE1 resulted in alteration of some immune response parameters but did not appear to result in increased survival duration. However, 2 dogs in the study achieved survival times in excess of 1 year. Weak replication of OC-CAVE1 in metastatic cells and delay of chemotherapy following CRAd treatment may contribute to the lack of immune response and improvement in survival time of the clinical patients.

## Teaser

Dog; Cancer; Osteosarcoma; Tumor immunology; Canine adenovirus 2

## 1 Introduction

Appendicular osteosarcoma (OSA) is a common neoplasm in dogs, with at least 8,000–10,000 cases diagnosed per year, accounting for 5–6% of all canine malignancies and approximately 80% of all canine bone neoplasms [1, 2]. The disease usually occurs in middle to older aged, large or giant breed dogs [1, 3, 4]. Current treatments consist of palliative amputation or limb-sparing techniques combined with platinum-based chemotherapy. Amputation alone results in a median survival time of approximately 6 months, which increases to approximately one year if chemotherapy is added, but most dogs still succumb to metastatic disease [2, 4, 5]. Therefore, a different strategy is needed to improve survival times by eradicating the remaining neoplastic cells and, in particular, focusing on eradication of micrometastatic disease.

Oncolytic viruses are a promising tool for cancer gene therapy. Adenoviral (Ad) vectors are among the most commonly used oncolytic vectors. Conditionally replicative adenoviruses (CRAd) are designed so that virus replicates only in the target tissue, thus ensuring specificity of any observed cytopathic effects (CPE) to those target cells. Replication of the CRAd in turn releases thousands of new virus particles which may go on to infect new tumor cells [6]. CRAds have also been proposed as a potent therapy for eradication of cancer stem cells (CSC) [7, 8]. CSCs are quiescent cells with a high tumorigenic potential and are resistant to many cancer therapies. Therefore, it has been proposed that CSCs are responsible for causing tumor micro-metastases and relapse [9].

A further benefit of oncolytic viruses in the treatment of cancer is that they may be able to enhance existing immunity to tumors [10]. *Listeria* vaccine induced HER2/neu + appendicular OSA-specific immunity, reduced the metastatic incidences, and increased the overall survival in spontaneous clinical canine patients [11]. Oncolysis due to CRAds can result in overcoming immune tolerance and generating an immune response to tumor cells [6, 12]. Immune cell recruitment to the tumor mass can result from both the lysis of the tumor cell and the release of cytokines from infected cells [10, 13]. It is well documented that there is a robust immune response to many tumors, evident by infiltrating immune cells including T cells, B cells, natural killer (NK) cells and dendritic cells (DCs) [10]. An increase in the number of DCs in the site of the tumor has been suggested as having beneficial anti-tumor activity and resulted in the development of DC based vaccine therapies for a number of cancers [13]. However, the tumor microenvironment is inherently immunosuppressive due to the presence of regulatory T cells (T-regs), which are present to varying degrees in different types of neoplasms. Circulating T-regs are increased in dogs with cancer and increased numbers of T-regs are associated with a poorer prognosis and decreased survival time in canine OSA patients [14].

Previously, we developed a canine CRAd (OC-CAVE1) based on canine adenovirus type 2 (CAV2) for use in canine OSA, [15]. CAV2 is commonly used as a vaccine against CAV1, the agent of canine hepatitis. The CRAd OC-CAVE1 was constructed by replacing the wild type promoter of the CAV2 E1A gene with the osteocalcin promoter that restricts its replication to canine OSA cells and causes CPE only in these cells [16]. OC-CAVE1 also shows significant anti-tumor activity against established canine OSA xenografts in a nude mouse model [16].

Safety of OC-CAVE1 was determined by injecting virus intravenously into 3 normal dogs and 3 transiently immunosuppressed dogs. All dogs had pre-existing anti-CAV2 antibody titers due to CAV2 vaccination. Immunosuppression was employed because it was hypothesized that an anamnestic response to CAV2 would result in faster elimination of virus and thus reduce ability of CRAd to infect metastatic cells. However, no change in circulating CRAd viral numbers was observed in immunosuppressed dogs when compared to non-immunosuppressed dogs and the only adverse event noted was a short-lived lymphopenia, consistent with viral infections [15]. Based on the data in normal dogs, a preliminary safety study was initiated to determine the safety of virus in 4 dogs affected with OSA (data unpublished). The longest-lived dog in this study (25 months) developed pulmonary nodules at 1 year after treatment. Histopathologic examination of the nodule showed caseous necrosis. While there are many possible causes of pulmonary caseous necrosis, an intriguing possibility was that this dog had an immune response to tumor, walling off metastatic tumor in the lung, perhaps in response to OC-CAVE1 infection and immune stimulation. The preliminary safety study was not designed to evaluate this question, thus the present study was initiated to further examine this hypothesis.

Given that OC-CAVE1 can be safely administered to dogs, can be specifically engineered to replicate only in tumor cells, may target CSCs, and more importantly potentially generate an enhanced immune response to tumor, we started an expanded pre-clinical study in canine OSA patients. Ten patients were fully enrolled over the 2-year study period. The findings from this pilot experiment, focused on the immunologic results of CRAd therapy for canine OSA, are summarized in this report.

## 2 Materials and methods

### 2.1 Patient admission and CRAD injection

A group of 11 client-owned canine patients with spontaneously occurring OSA were enrolled in the study (Table 1). Written informed consent to enroll the animals was obtained from the owners of the animals. Enrollment of animals was approved by the Clinical Research Review Committee of the College of Veterinary Medicine (CRRC) and the Auburn University Institutional Animal Care and Use Committee (IACUC). All patient dogs were pre-evaluated and had confirmed the diagnosis of OSA before being accepted into the study. All dogs were pre-vaccinated for CAV. All dogs except Patient 1 underwent limb amputation at the Wilford and Kate Bailey Small Animal Teaching Hospital. Patient 1 underwent limb-sparing surgery. Following 3 days' recovery in the ICU after limb amputation surgery, 2 × 10^12^ CRAD virus particles of OC-CAVE1 (generously gifted by Dr. David T Curiel and Dr. Igor Dmitriev) were injected into the dog's cephalic vein over approximately 5 min. Viral stocks were stored at -85 °C and thawed on ice immediately prior to use. Virus doses were diluted to a final volume of 5 ml in ice cold Phosphate buffered saline (PBS). The dogs were isolated and monitored for signs of shock or other manifestations of toxicity for 6 days, using VCOG criteria [17]. This included monitoring the dogs' vital signs by hospital staff and veterinary students (body temperature, pulse rate, and respiratory rate) at 15 min pre-injection and 15 min, 30 min, and at 1, 2, 4, 12, 24, 36, 48, 60, 72, 84, 96, 108, 120, 132, and 144 h post-injection. Blood (2ml) and free catch urine (1ml) and feces were collected every 12 h post-injection for 6 days for PCR determination of CRAd concentration in these materials. Blood (2ml) samples were also taken at 48 h and 144 h post-injection for complete blood count (CBC) and blood chemistry analysis by the Auburn University Clinical Pathology Laboratory. At the end of the isolation period, the dogs were released to their owners. Dogs returned to the hospital at approximately monthly intervals for examination and the final outcome of the dogs was determined by either examination of the clinical record or direct contact with the owner.

**Table 1:** Demographics of all the patients enrolled in the study.

Patient	Breed	Age	Sex/spayed-neuter	Weight (kg)	Location	Suspected Metastasis at the time of CRAd Administration	Surgery	Suspected Metastasis at the Terminal Stage	Cause Of Death
1	Great Dane	8	Female/spayed	52.1	Left Distal Ulna	Not Diagnosed	Partial Distal Ulnectomy	Keratin Cyst Right Lumbar Area	Euthanasia for OSA
2	Mixed Breed	8	Male/neutered	45.17	Left Proximal Humerus	Not Diagnosed	Left Forelimb Amputation	None	Euthanasia not for OSA
3	Great Dane	7	Female/spayed	48.8	Left Proximal Humerus	Not Diagnosed	Left Forelimb Amputation	Unknown	Unknown
4	Rottweiler	8	Female/spayed	44	Left Distal Tibia	Not Diagnosed	Left Hindleg Amputation	Unknown	Unknown
5	Boxer	13	Male/neutered	31.75	Right Distal Femur	Not Diagnosed	Right Hind Limb Amputation	Caudal Abdominal Mass And Pulmonary Nodule Identified	Euthanasia for OSA
6	Greyhound	7	Male/neutered	27.4	Right Distal Radius	Not Diagnosed	Right Hind Limb Amputation	Skin Nodules	Euthanasia for OSA
7	Rottweiler	8	Female/spayed	31.48	Right Distal Radius	Suspected Pulmonary Nodule	Right Hind Limb Amputation	Pulmonary Metastasis and Possible Rib Metastasis	Euthanasia for OSA
8	Shar-Pei	9	Male/neutered	17.78	Left Front Limb	Suspected Pulmonary Metastasis and Right Ear Sarcoma	Left Forelimb Amputation	Pulmonary Metastasis	Euthanasia for OSA
9	Greyhound	9	Male/neutered	28.1	Right Proximal Tibia	Not Diagnosed	Mid Femoral Amputation of Right Hind	Suspect Pulmonary Metastasis	Euthanasia for OSA
10	Australian Shephard	13	Female/spayed	19	Left Proximal Humerus	Suspected Metastasis to Regional Lymph Nodes	Left Forelimb Amputation	Suspected Metastasis to Regional Lymph Nodes	Suspected Embolism
11	Greyhound	16	Female/spayed	24.5	Left Distal Femur	Not Diagnosed	Left Hind Limb Amputation	Not Diagnosed	Surgical Complications

Patient information was collected from the electronic medical record at College of Veterinary Medicine at Auburn University. This information was recorded throughout the study. Patients 3 and 4 did not return to College of Veterinary Medicine at Auburn University for terminal stage care, and therefore, there is limited or no information regarding their terminal stage diagnosis and condition.

### 2.2 Autologous osteosarcoma cell culture

In order to generate autologous tumor cells for immunological analyses, the amputated limb was collected from surgery and small tumor pieces were removed and diced into 1 mm cubes. The remaining tumor mass was submitted for histopathological confirmation of the diagnosis of OSA. The tumor cubes were cultured in complete DMEM (Dulbecco's Modified Eagle's Medium, Corning) with penicillin (300 IU/ml, Corning), streptomycin (300 ug/ml, Corning), amphotericin B (1.5ug/ml, Corning), and 20% FBS (fetal bovine serum, Sigma) at 37 °C in 6 well plates as tumor explants. The media was changed weekly until cells were 80–90% confluent. Adherent cells were passaged two to four times before cryopreservation. Cells were cryopreserved in Freezing medium (10% DMSO + complete DMEM media) by gradually cooling the cells to -80 °C. Cryopreserved cells were stored in liquid nitrogen. Cryopreserved OSA cells were thawed at 37 °C. OSA cells were cultured in complete DMEM (Dulbecco's Modified Eagle's Medium, Corning) with penicillin (100 IU/ml, Corning), streptomycin (100 ug/ml, Corning), amphotericin B (0.5ug/ml, Corning), and 20% FBS (fetal bovine serum, Sigma) at 37 °C.

### 2.3 Blood draw and PBMC isolation

50 ml blood was drawn from the cephalic vein of all enrolled dogs prior to CRAd administration (day 0) and at 4 weeks after virotherapy. Blood was collected in EDTA tubes to prevent coagulation. 2ml blood was saved for monitoring CRAD levels pre and post CRAD injection by quantitative PCR. Blood was centrifuged at 450 x g for 30 min at room temperature. After centrifugation, the top plasma layer was collected and saved at -80 °C. The middle buffy coat layer was collected and transferred to 15 ml conical tubes (VWR). 2 ml of 1X PBS (Corning) was slowly added to the buffy coat. 5 ml of Histopaque 1077 (Sigma) was layered below the buffy layer and PBS mix. The layered suspension was centrifuged at 700 x g for 30 min. The cellular middle white layer was extracted post-centrifugation and collected in a 15ml conical tube. An equal amount of 1X PBS was added and the cells centrifuged at 700 x g for 10 min. The supernatant was removed, and the white cell pellet was re-suspended in 5ml 1X PBS and centrifuged again at 700 x g for 10 min. After centrifugation, the supernatant was removed and the PBMC (Peripheral Mononuclear Blood Cells) cell pellet was re-suspended in complete RPMI (Roswell Park Memorial Institute medium, Corning) with penicillin (100 IU/ml, Corning), streptomycin (100 ug/ml, Corning), amphotericin B (0.5ug/ml, Corning) and 10% FBS (Fetal bovine serum, Sigma). PBMCs were counted and then cryopreserved as described above in freezing media (10% DMSO + Complete RPMI media) for further experiments.

### 2.4 Anti-CAV2 titers

Plasma collected from blood drawn before and post-CRAD administration was sent to the Virology Laboratory at College of Veterinary Medicine, Auburn University for CAV-2 neutralizing antibody titers. CAV2 titers were determined by serum neutralization using 100 TCID of a CAV2 vaccine derived virus on Fieldsteel canine kidney cells.

### 2.5 DNA extraction, Primer Design, and Quantitative PCR

DNA was extracted from blood, feces, and urine samples using QIAamp DNA mini kit, QIAamp Fast DNA Stool mini kit, and QIAamp Viral RNA mini kit respectively, according to the manufacturer's instructions. DNA quality was assessed using nanodrop spectrophotometer. The concentration of DNA was determined by absorbance at 260 nm. The sequences to amplify CAV2-E1B gene were the forward primer GCTTGCTACATTATTGGTAA, reverse primer CAAGGTGTTTCTTTCACTAA, and probe 6FAM-CTAACCTGCCTGCTGGAGAA-TAMRA. TaqMan primers and probes were designed by the DNA Star software and commercially synthesized (Eurofins Genomics). The qPCR master mix was designed with a final volume of 15 ul per reaction containing 1X Sso Advanced™ Universal Probes Supermix (BioRad), 250 nM forward primer, 250 nM reverse primer, and 250 nM probe. For the assay, known amounts of OC-CAVE1 template DNA (10^8^, 10^6^, 10^4^, and 10^2^ copies) were amplified to generate a standard curve for quantification of the OC-CAVE1 copy numbers in the experimental samples. Five microliters (5ul) of the sample was added to 15ul of PCR master mix in each reaction well. PCR was performed in 96 well plates. Thermal cycling conditions were 3 min at 95 °C and 40 cycles of 5 s at 95 °C and 10 s at 54 °C. qPCR was performed using a Bio-Rad iCycler iQ Multicolor Real-Time PCR machine. PCR products were purified using GeneJet gel extraction kit (Thermo) according to the manufacturer's instructions and identity was confirmed by sequencing (Eurofins MWG Operon).

### 2.6 Protein extraction and western blot

Cryopreserved primary OSA cells were thawed and cultured in T75 culture flasks in complete DMEM media (as described above). At approximately 80% confluence, the cells were washed with cold 1X PBS. 1ml RIPA (Radioimmunoprecipitation assay) lysis and extraction buffer (Thermo Scientific) along with 1X Halt Protease and Phosphatase Inhibitor Cocktail (Thermo) was added to the cells. Cells were lysed using one cycle of freeze/thaw and passage through 21-gauge and 23-gauge needles. All steps were performed on ice. The cell lysate was centrifuged at ~14,000 x g for 15 min at 4 °C. The supernatant was transferred to a new tube and stored at -20 °C. Supernatants were assayed for protein concentration using a Bicinchoninic Acid Assay (BCA200 protein assay kit, Pierce). For normalization purposes equal starting volumes of protein cell lysates, extracted from OSA cells, were incubated in boiling water in Lane Marker Reducing Sample Buffer (Pierce) for 10 min before loading onto a polyacrylamide gel (4–20% and 8–16% precise™ protein gels, Pierce) along with Kaleidoscope markers (range 10–250 kDa, BioRad). Electrophoresis was run in Tris–HEPES–SDS running buffer (Pierce) for 1 h at 100 V and proteins were transferred to PVDF membranes (Immobilone1-P Transfer Membrane, Millipore) using transfer buffer (25mM bicine, 25mM Tris-base, 10% Methanol) for 1 h at 100 V. The membrane was washed with 1X wash buffer (1X PBS and 0.1% v/v Tween 20). The membrane was blocked with blocking buffer (5% non-fat milk powder dissolved in 1X wash buffer) for 2 h at room temperature. The membrane was incubated with autologous blood plasma (1:100) overnight at 4 °C. The membrane was washed with wash buffer 3 times for 10 min each at room temperature. The membrane was incubated with goat anti-dog IgG (H + L) -HRP secondary antibody (1:10,000; Sigma) for 1 h at room temperature. The membrane was washed again 3 times for 10 min each at room temperature. Western blot analysis was performed using WesternSure Chemiluminescence Reagent (LICOR) according to the manufacturer's instructions. Membranes were scanned digitally using C-Digit Blot Scanner (LICOR).

### 2.7 Cytotoxic T lymphocyte (CTL) assay

CTL assays were performed against cryopreserved primary autologous OSA cells isolated from each individual patient dog tumor according to previous published assay [18]. 5 × 10^3^ target autologous OSA cells/well were plated in 96 well plate on day 1 in complete RPMI (Corning) with penicillin (100 IU/ml, Corning), streptomycin (100 ug/ml, Corning), amphotericin B (0.5ug/ml, Corning), and 20% FBS (fetal bovine serum, Sigma) at 37 °C in T75 cell culture flasks. The next day, 15ul ^51^Cr mix (98 μCi in 302μl and 20μl FBS; 3.5uCi/5X10^3^ cells) added directly to each well for 2 h at 37 °C. ^51^Cr-media was removed and cells were washed once with RPMI complete media, and post-incubated in the RPMI-1640 medium for 45 min at 37 °C twice to reduce background. Cryopreserved PBMCs were thawed at 37 °C and were rested for 1hr on ice in complete RPMI media before adding to the target autologous OSA cells. Effector PBMCs were added in three different ratios (1:25, 1:50, and 1:100) in three sets of triplicate wells to measure experimental isotope release for 16h to ensure assay reliability as previously described for canine lymphocytes [19, 20, 21, 21]. Effector PBMCs were not added in one set of triplicate cells to measure spontaneous isotope release. Cells in another set of triplicate wells were lysed using lysis buffer (1XPBS + 10% Triton-X) to measure maximal isotope release. Supernatants were counted for released radioactivity by liquid scintillation counting in triplicate. Corrected percent lysis was calculated to determine relative cytotoxic T-lymphocyte (CTL) activity. Cell mediated immunity was calculated using following formula:

%CMI (specific lysis) = (EIR – SIR/ MIR – SIR) X 100

EIR (experimental isotope release) = Counts per minute (CPMs) released by CTL-specific activity in wells with labeled target cells lysed by PBMCs effector cells.

SIR (spontaneous isotope release) = CPMs released in the absence of any lysis (negative control) in labeled target cells with no effector cells.

MIR (maximal isotope release) = CPMs released by 10% Triton X-100 lysis of labeled target cells.

Flow Cytometry for Humoral Anti-Tumor Activity, Levels of Antigen Presenting Cells and Regulatory T Cells.

#### 2.7.1 Humoral anti-tumor activity

Autologous OSA cells were cultured in T75 flasks in complete DMEM media (as described above). Cells were harvested using 1X trypsin (Corning). 2.5 × 10^5^ cells/sample were blocked with blocking buffer (1X PBS + 10% FBS + 0.2ug anti-mouse CD16/CD32; Thermo, Cat# 14-0161-82) for 1 h at room temperature. Cells were centrifuged and washed once with wash buffer (1X PBS + 1% BSA). Cells were incubated in autologous plasma (pre- and post-injection) for 1 h at room temperature. Cells were washed once with wash buffer and incubated in secondary antibody, goat anti-dog IgG-FITC (Santa Cruz Biotechnology) for 1 h at room temperature. Cells were washed again once with wash buffer and analyzed for humoral anti-tumor activity by flow cytometry.

#### 2.7.2 Antigen presenting cells

Frozen PBMCs were thawed at 37 °C and were rested for 30 min at 4 °C (with caps cracked open). Cells were blocked in dog block (1X PBS + 10%NDS + canine Fc Receptor binding inhibitor; Thermo Cat# 14-9162-42) for 30 min at room temperature. Cells were washed in 1X PBS and stained with fixable viability dye (Biolegend; Cat# 423111) and incubated for 30 min at room temperature. Cells were washed again with 1X PBS and stained with anti-canine CD11c antibody (Biorad, Cat# MCA1778S) tagged with Zenon ® Alexa Fluor® 700 (Thermo, Cat# Z25011) and incubated for 30 min at room temperature. Cells were washed and analyzed for CD11c positive cells by flow cytometry.

#### 2.7.3 Regulatory T cells

Frozen PBMCs were prepared as described for antigen presenting cells, above. Cells were washed in 1X PBS and stained with fixable viability dye (Biolegend; Cat# 423111) and incubated for 30 min at room temperature. Cells were washed again with 1X PBS and stained with anti-canine CD4:Alexa Fluor® 488 antibody (Biorad, Cat# MCA1038A488) at 1:25 dilution and incubated for 30 min at room temperature. Cells were washed and fixed with Foxp3/Transcription Factor Fixation/Permeabilization buffer (Thermo, Cat# 00-5521-00) and incubated overnight at 4 °C. Cells were washed with 1X permeabilization buffer and stained with 0.5 ug of anti-mouse/Rat FoxP3 PE antibody (Thermo, Cat# 12–5773) for 30 min at room temperature. Cells were washed with 1X permeabilization buffer and analyzed for CD4+FoxP3+ cells by flow cytometry.

### 2.8 Statistics

Statistics were performed on the CAV2 titers, CTL assays, and all flow cytometry data. All the experiments were done in triplicates, except CAV2 titers, which were assessed only one time. Statistical significance was determined using paired t-test. The significant threshold was set at p < 0.05.

## 3 Results

### 3.1 Virus quantitation

Samples of urine and feces were collected at every 12 h post-virus-injection for viral DNA quantification by PCR. Samples of blood were collected before virus injection, every 12 h after virus injection, and 4 weeks post-injection, just prior to the initiation of chemotherapy. Viral DNA was weakly positive in urine samples of patients 1, 2, 4, and 8 in 50% or more of the samples collected (Table 2). Viral DNA was transiently expressed in 2 urine samples from patients 3 and 7. Dogs 5, 6, 9, and 10 showed no detectable shedding in the urine.

**Table 2:** Quantitation of OC-CAVE1 CRAd genomes in clinical patient's urine using qPCR.

Hours Post Virus-Injection	Patient 1	Patient 2	Patient 3	Patient 4	Patient 5	Patient 6	Patient 7	Patient 8	Patient 9	Patient 10
12	4.1	0.53	NS	5.52	0	0	0.50	1.29	NS	0
24	7.4	0.51	0	0.84	0	0	0	0.55	0	0
36	2.8	0.98	NS	0	0	0	0	2.47	0	0
48	3.0	0.91	0	2.1	0	0	0	0	0	0
60	3.6	0.35	NS	0.56	0	0	0	0	0	0
72	9.4	0	0	0.52	0	0	0	4.09	0	0
84	0	0	0	0	0	0	0.83	0	NS	0
96	5	0.23	0.029	0.49	0	NS	0	0	0	0
108	8.6	1.32	NS	1.47	0	0	0	0	0	0
120	NS	0.71	0	0.75	0	0	0	0.70	0	NS
132	0	0.98	0	0.32	0	0	0	0.87	0	0
144	0	1.03	0.005	0	0	0	0	0	0	0

Values are reported as viral copies/ul of sample. A standard curve of known amounts of OC-CAVE1 CRAd template DNA (108, 106, 104, and 102 copies) was used for the quantification reference. NS - no sample collected.

Viral DNA was present in occasional fecal samples including a single time point in dogs 1, 2, 8, and 9 at 24hrs, 108 h, 36 h, and 48 h respectively (Table 3). Fecal samples from dog 3 were positive at two, disconnected, time points (12 h, 96 h). Fecal samples from dogs 4 and 10 were positive at three time points. In both cases, two of these time points were sequential while the third was separated from the others by at least one time point. Dogs 5, 6, and 7 showed no detectable viral genomes in their feces.

**Table 3:** Quantitation of OC-CAVE1 CRAd genomes in clinical patient's feces using qPCR.

Hours Post virus-Injection	Patient 1	Patient 2	Patient 3	Patient 4	Patient 5	Patient 6	Patient 7	Patient 8	Patient 9	Patient 10
12	19.06	0	1.99	0	0	NS	0	0	NS	0
24	0	0	0	0	0	NS	0	NS	3.44	0
36	0	0	0	0	0	NS	0	7.58	0	0
48	0	0	0	0	0	0	0	NS	0	0
60	0	0	0	0	0	0	0	0	NS	NS
72	0	0	0	0	0	NS	0	0	0	NS
84	0	0	0	0	NS	NS	0	0	0	0.312
96	0	0	3.84	0	0	0	0	NS	0	4
108	0	12.26	0	0.39	0	NS	0	0	NS	NS
120	0	0	0	0	0	NS	0	0	0	NS
132	0	0	0	2.6	0	NS	0	0	NS	11.1
144	0	0	0	0.37	0	NS	0	0	0	NS

Values are reported as viral copies/ug of sample. A standard curve of known amount of OC-CAVE1 CRAd template DNA (108, 106, 104, and 102 copies) was used for the quantification reference. NS - no sample collected.

**Figure 1:**
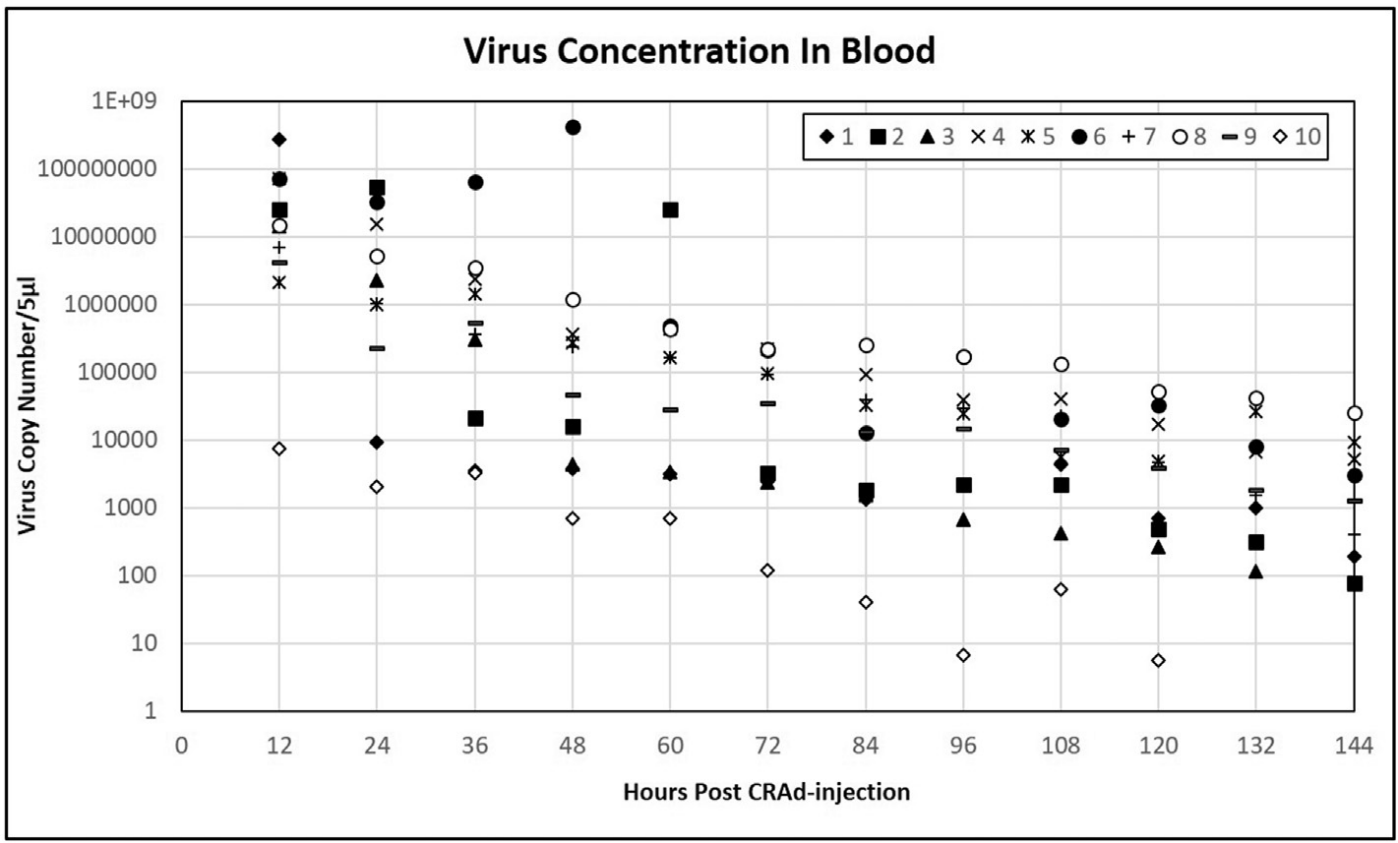
Quantitative RT-PCR analysis of OC-CAVE1 CRAd genomes in clinical patient's blood. Absolute quantification of CAV2-E1B genomes was determined using a standard curve.

Viral DNA was not detected in blood samples from any dogs before virus injection. Viral DNA was detected in the circulation of all dogs following administration and generally decayed in a logarithmic manner over the course of the subsequent 6 days (144 h) (Table 4; Figure 1). Although input doses were identical, initial viral concentrations in blood post-CRAd administration ranged over more than four orders of magnitude. Initial viral concentrations in blood post-CRAd injection are not related to the weight of dogs. While most dogs showed small amounts of variability in the amount of reduction of circulating virus, two dogs showed clear peaks in viral copy number in their circulation. Dog 2 showed a single elevated sample at 60 h post injection, while dog 6 showed an elevation in viral DNA in circulation beginning at 36 h, peaking at 48 h and returning to the normal decay curve at 60 h. Virus remained detectable in the circulation of eight of the ten dogs through 144 h post injection, with only dogs 3 and 10 showing no detectable circulating viral genomes prior to the end of sampling (132, 144 h), Subsequently, no virus was present in the blood of any dog 4 weeks post-virus-injection, just prior to the start of chemotherapy.

**Table 4:** Quantitation of OC-CAVE1 CRAd genomes in clinical patient's blood using qPCR.

Hours Post virus-Injection	Patient 1	Patient 2	Patient 3	Patient 4	Patient 5	Patient 6	Patient 7	Patient 8	Patient 9	Patient 10
0	0	0	0	0	0	0	0	0	0	0
12	53800000	5140000	2820000	14520000	426000	14160000	1368000	2940000	850000	1484
24	1880	10620000	458000	3060000	199600	6560000	206000	1042000	46200	412
36	690	4160	60200	464000	288000	12860000	73600	706000	107600	642
48	752	3220	874	71200	54800	85000000	46800	240000	9340	137
60	630	5060000	682	86600	33400	96000	32400	86200	5540	140
72	480	656	470	43400	19480	42200	18220	44400	6920	23
84	262	366	306	18320	6440	2600	7680	51400	2680	8
96	446	444	133	7720	4800	34600	5880	34600	2960	1
108	870	442	84	8140	1108	4080	4520	26600	1416	12
120	138	97	53	3460	964	6540	942	10560	778	1
132	200	63	22	1340	5200	1576	308	8280	366	0
144	38	15	0	1048	1862	612	80.2	5040	252	0
4 weeks	0	0	0	0	0	0	0	0	0	0

Values are reported as viral copies/ul of sample. A standard curve of known amount of OC-CAVE1 CRAd template DNA (108, 106, 104, and 102 copies) was used for the quantification reference.

### 3.2 Anti-CAV2 titers

**Figure 2:**
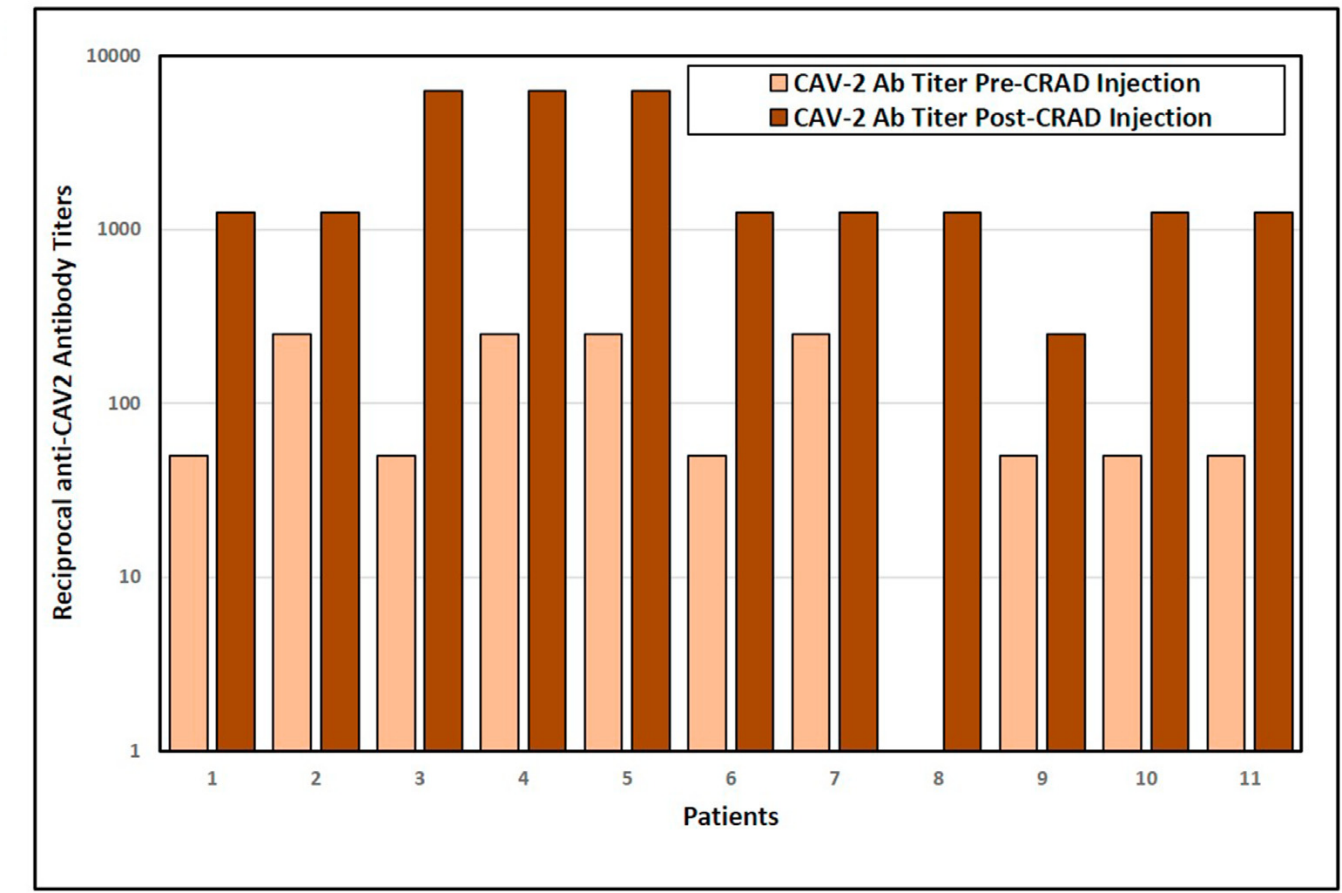
CAV-2 antibody reciprocal titers. Reciprocal anti-CAV2 circulatory neutralizing titers are compared between plasma samples collected before virus-injection and 4-weeks post-virus injection.

Pre-existing immune responses to CRAds have been raised as a possible concern, due to the potential for antibody to CAV2 to remove CRAd virus from circulation. All of the dogs enrolled in the study had been vaccinated with CAV2 within the previous 3 years. Plasma samples taken prior to injection and four weeks after CRAD injection were assessed for CAV-2 neutralizing antibody titers (Figure 2). All dogs, except dog 8, showed existing anti-CAV2 titers prior to administration of the CRAd. All dogs showed significant increases (Paired T-test; 3.36, P-value = .0072) in CAV-2 antibody titers 4 weeks after CRAD-injection. The fold increase in CAV-2 antibody titers between pre-and post-injection varied from a 5-fold increase in dogs 2, 7, and 9 to a 125-fold increase in dog 3 (Figure 2).

### 3.3 Humoral immune responses to tumor

Humoral immune responses to autologous tumor were examined by two different approaches, Western blot and flow cytometry.

### 3.4 Western blot of autologous tumor cell lysates with patient serum

**Figure 3:**
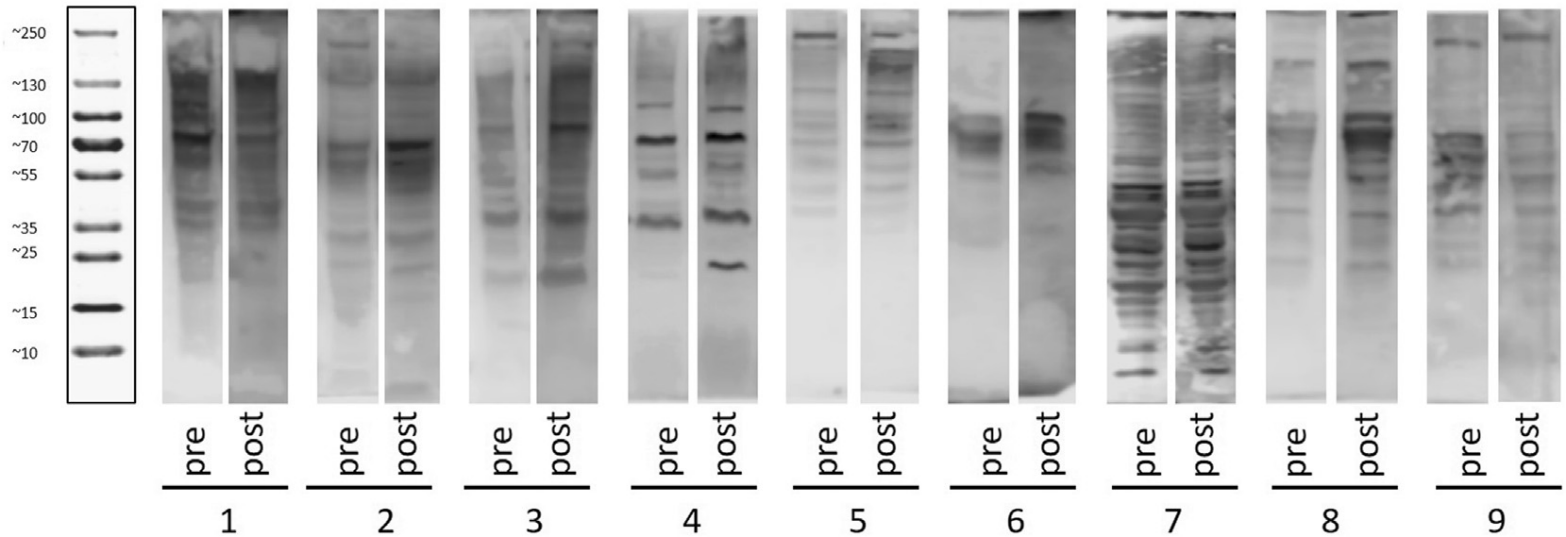
Western-blot analysis to compare circulating antibodies generated against autologous tumor antigens pre- and post-CRAd administration. For each patient, autologous blood plasma was used as primary antibody and goat anti-dog IgG-HRP antibody was used as secondary antibody. The blots were scanned using C-Digit Blot Scanner (LICOR).

To examine whether OC-CAVE1 CRAd administration generated antibodies against tumor antigens, we compared serum antibody reactivity against autologous tumor cell lysates between pre-administration and 4 weeks post-administration patient samples. Tumor cell lysates from each patient were subjected to polyacrylamide gel electrophoresis, blotted onto membranes, probed with autologous serum, and visualized with an antibody against canine IgG. Numerous protein bands were visualized in all of the pre-administration samples, indicating that all of the dogs in the study had pre-existing circulating antibodies to proteins present in their tumors (Figure 3). Numerous bands were also visualized with post-administration serum in every dog. Many of the bands seen were present in both the pre- and post-administration samples. However, some of the visualized bands were of higher intensities in blots probed with post-administration serum. Examples of this include a band in dog 2 at approximately 70 kDA (kilodaltons), a band in dog 4 at approximately 20–25 kDA, and a band in dog 6 at approximately 100 kDA. Some bands appear in similar locations across multiple different dogs, indicating the potential that one or more common antigens are being recognized repeatedly.

### 3.5 Autologous anti-tumor IgG response induced post-vaccination

**Figure 4:**
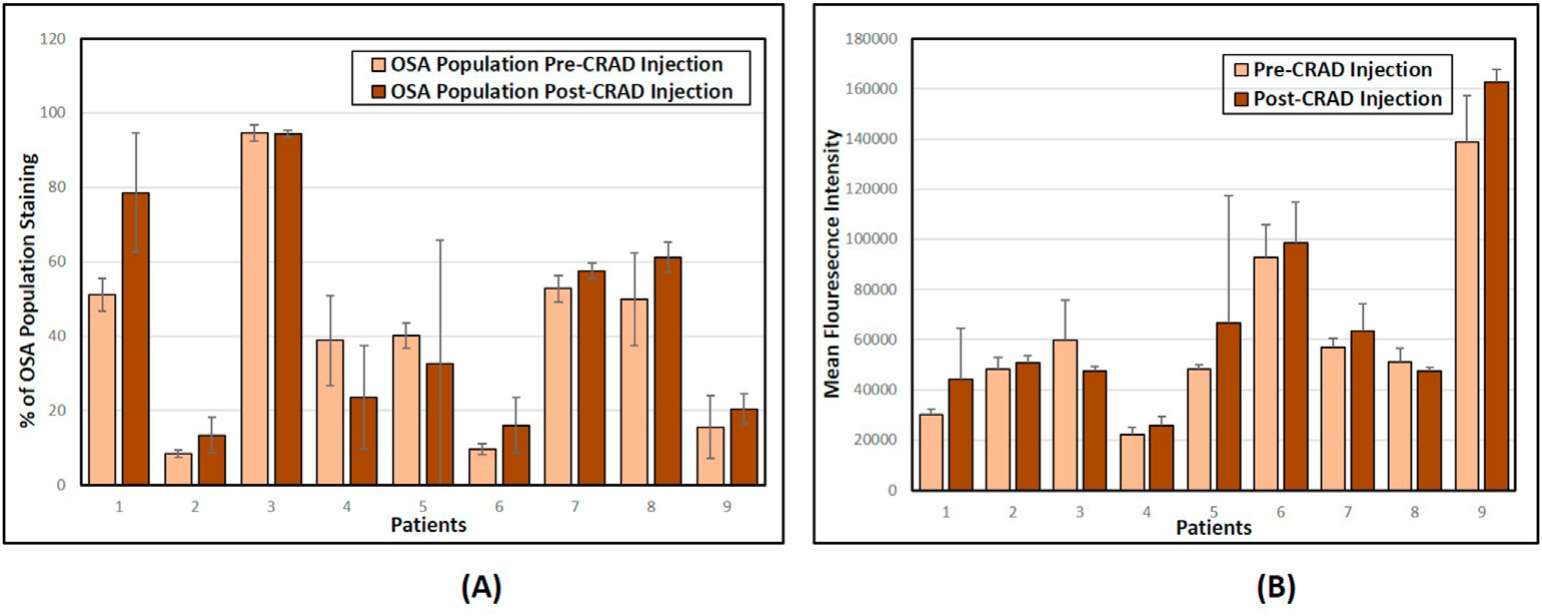
Flow cytometric quantification of the humoral response to autologous OSA cells. Autologous cells from each patient were incubated with their own plasma collected before and 4 weeks after CRAd administration. Secondary antibody used was anti-dog IgG-FITC. (A) The graph demonstrates the percent of the population stained by circulatory antibodies for each patient pre- and post- CRAd administration. (B) The graph demonstrates the mean fluorescence intensity of binding of circulatory antibodies to autologous cells for each patient pre- and post- CRAd administration. Analysis was done using Accuri flow cytometry software. Error bars represents standard deviation.

In order to quantify both the intensity of the anti-tumor IgG response as well as the proportion of tumor cells recognized by that response, we developed a flow cytometric protocol to quantify tumor-specific IgG responses against autologous tumor cells. Autologous plasma from each dog, obtained pre-administration and 4 weeks post-administration, was used as the primary antibody to label autologous tumor cells. An anti-dog IgG secondary antibody labeled with FITC was used to visualize the labeled cells. The anti-tumor IgG response against autologous tumor cells showed an increased number of cells staining positive in one patient, dog 1, after treatment (Figure 4A). IgG response against autologous tumor cells in the remaining dogs did not significantly increase post-virus administration (Paired T-test; -0.98, P-value = .3524). The mean fluorescence intensity of labeling was also examined to determine if the amount of antibody binding to individual cells had increased (Figure 4B). The mean fluorescence intensity did not significantly increase in the patients' post-administration (Paired T-test; -1.52, P-value = .1622).

### 3.6 Levels of circulating dendritic cells pre- and post-administration

**Figure 5:**
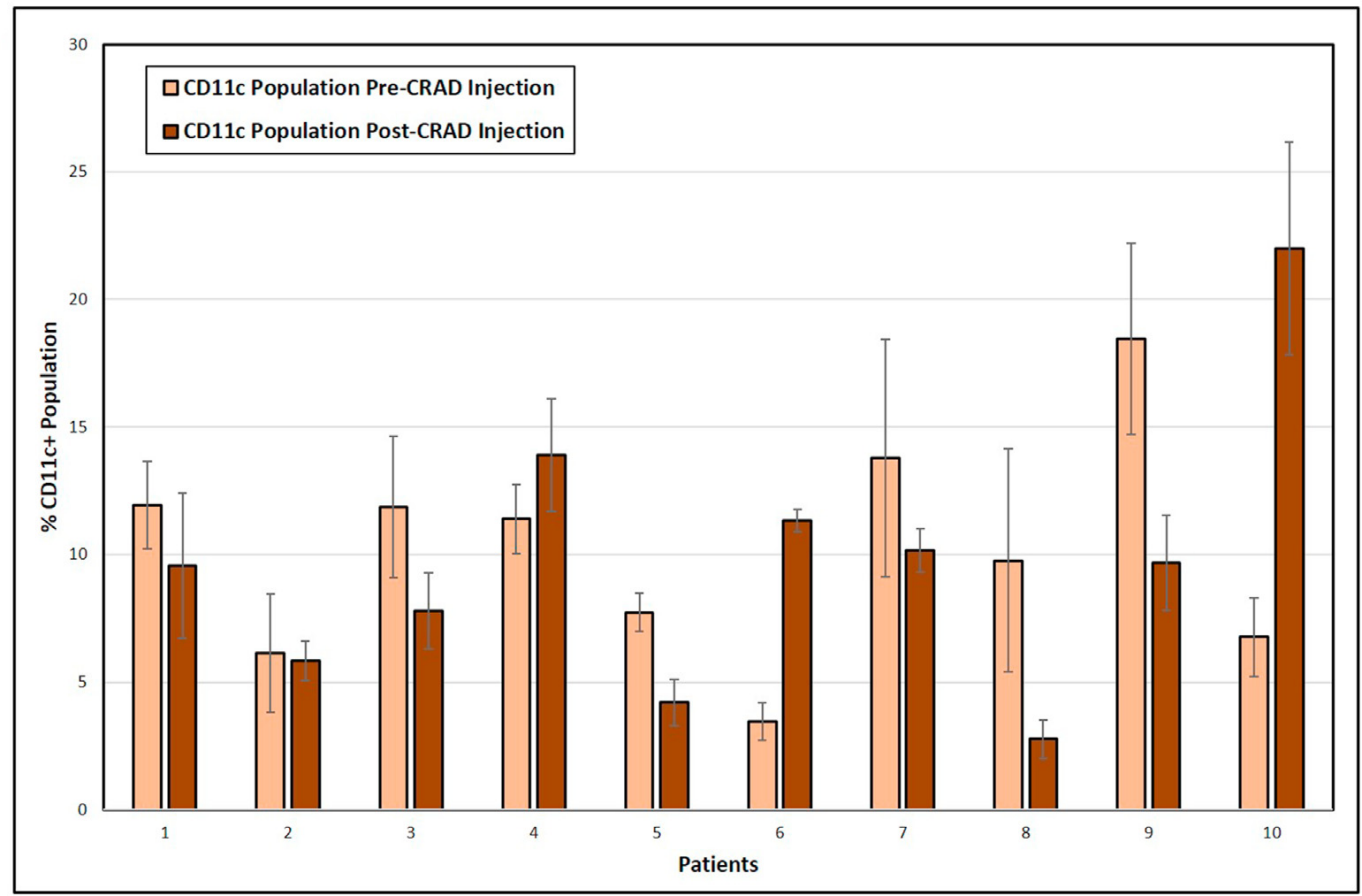
Flow cytometric quantification of circulatory CD11c + cells in Osteosarcoma patients. Blood was collected from patients and PBMCs were isolated and cryo-preserved before and after 4 weeks of CRAd administration. The PBMCs were stained for viability and with anti-canine CD11c antibody tagged with Zenon ® Alexa Fluor® 700. The graph demonstrated the percent of population positive for CD11c staining before and after CRAd injection. Analysis was done using Accuri flow cytometry software. Error bars represents standard deviation.

Dendritic cells, as defined in the dog by a population of cells positive for CD11c, are a critical component in presenting antigen. The presence of dendritic cells in the circulation reflects the antigen presentation status of the patient. The circulating CD11c-positive population in each patient dog was quantified by flow cytometry (Figure 5). There was an increase in the CD11c + population in patients 6 and 10. Interestingly, dogs 8 and 9 showed large decreases in their circulating CD11c population after CRAd administration while dogs 1, 3, 5, and 7 showed smaller decreases. However, as a group there was no significant change in CD11c + cells in patients post-administration (Paired T- test; 0.74, P-value, .4770).

### 3.7 Levels of regulatory T cells pre- and post-administration

**Figure 6:**
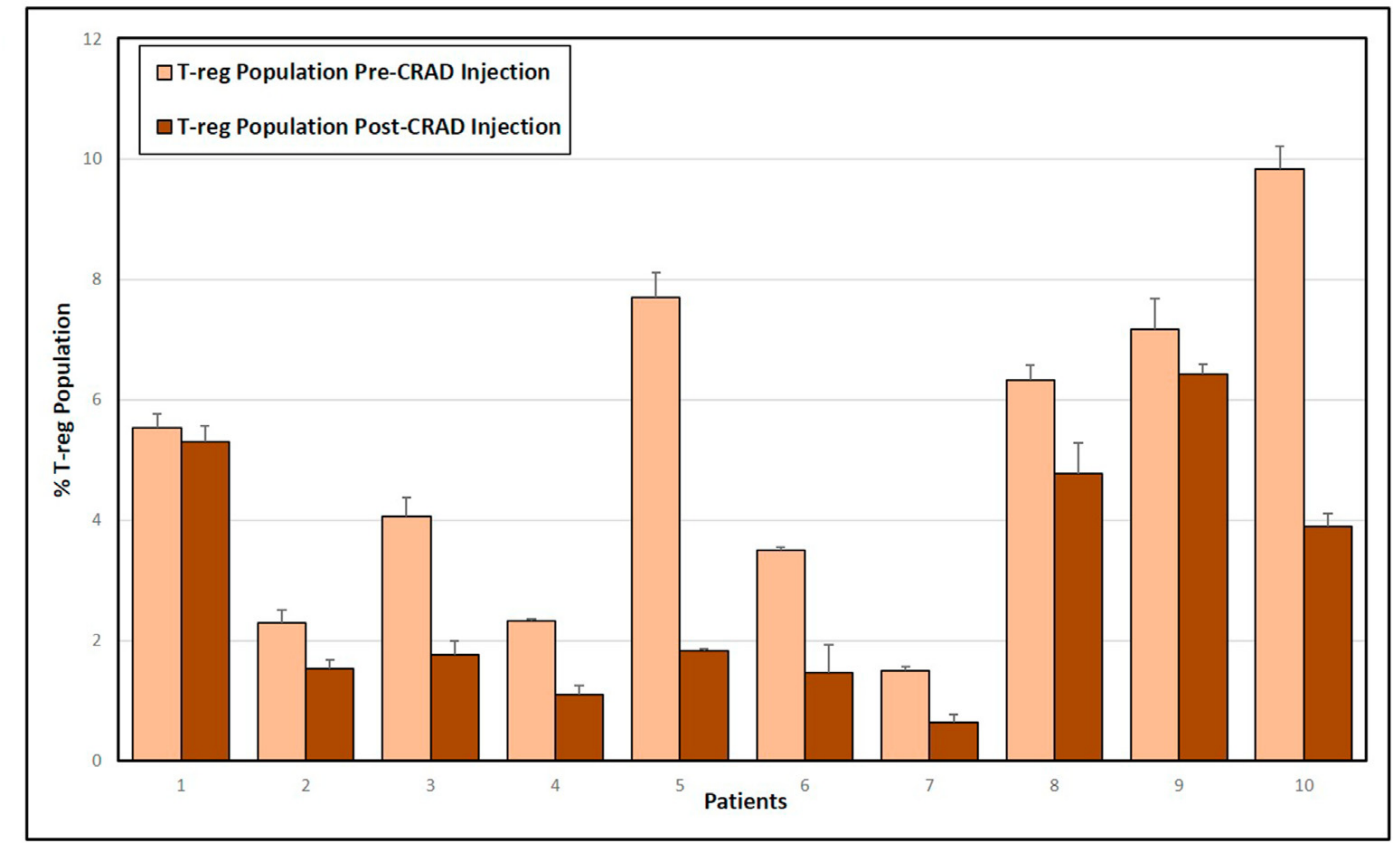
Flow cytometric quantification of regulatory T cells in Osteosarcoma patients. Blood was collected from patients and PBMCs were isolated and cryo-preserved before and 4 weeks after CRAd administration. The PBMCs were stained for viability and with anti-canine CD4:Alexa Fluor® 488 antibody and anti-mouse/Rat FoxP3 PE antibody. The graph demonstrated the percent of circulatory CD4+FoxP3+ cells present before and after CRAd injection. Analysis was done using Accuri flow cytometry software. Error bars represents standard deviation.

Regulatory T-cells have been implicated in the suppression of anti-tumor immune responses. The circulating population of T-regs, identified as CD4+FoxP3+, was determined by flow cytometry prior to administration of the CRAd and 4 weeks post-administration (Figure 6). The percentage of peripheral T-reg cells decreased significantly in patients post-CRAd administration (Paired T- test; 3.06, P-value, .0120).

### 3.8 Cytotoxic activity of tumor antigen-specific T cells against autologous tumor cells

**Figure 7:**
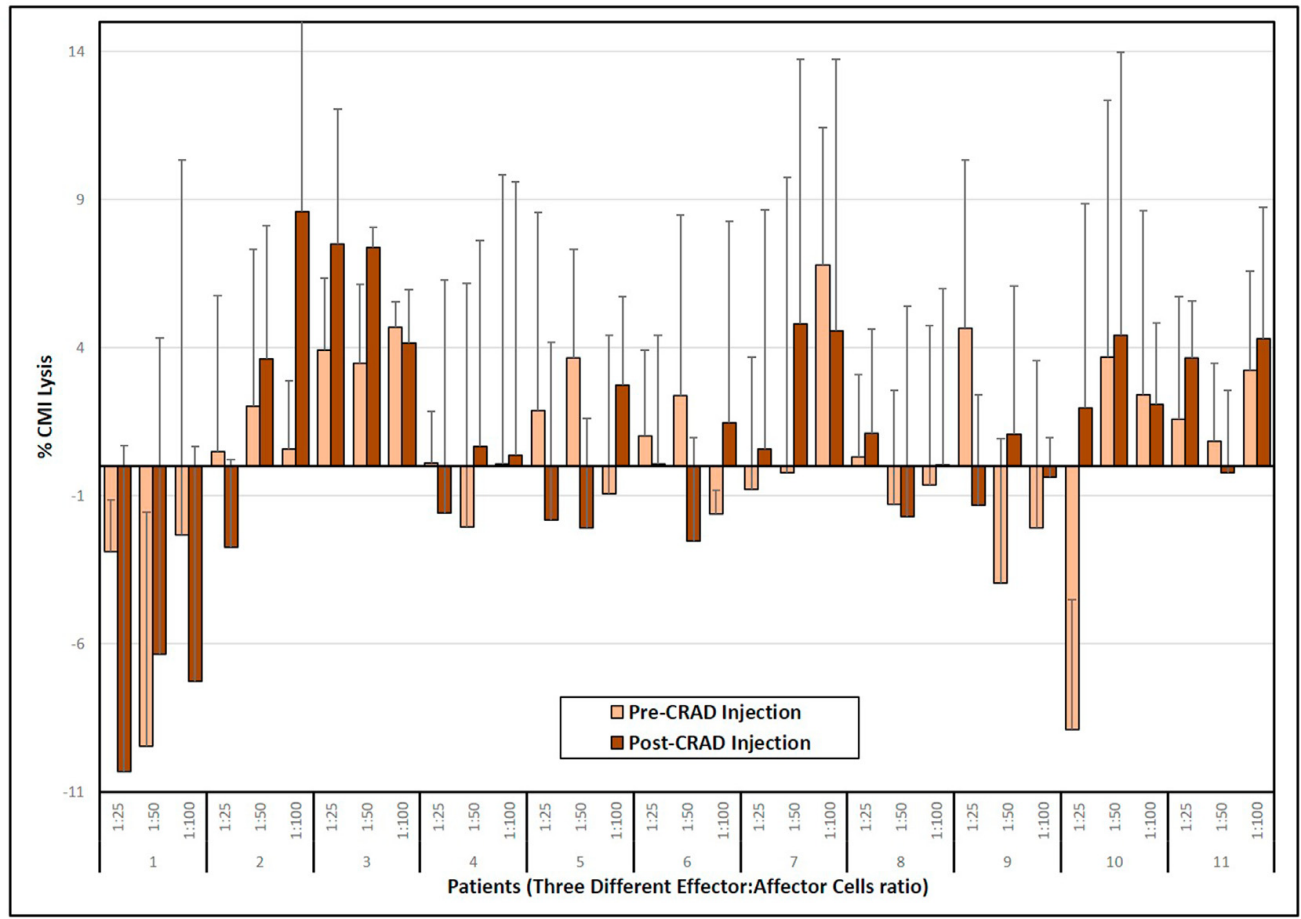
Cell mediated immunity of self-circulatory T-cells against autologous tumor cells. PBMCs were isolated and cryopreserved before and after 4 weeks of CRAd injection. Cytotoxic T lymphocyte (CTL) assays were performed by standard 51Cr-release, using PBMCs cultured with cryopreserved primary autologous OSA cells isolated from each individual patient dog tumor. CTL activity for each patient was measured in triplicate. CTL activity was calculated as percent-specific lysis (%CMI) and total releasable isotope availability for each PBMC:OSA target cell ratio for each patient (1:25, 1:50, and 1:100). All error bars represent standard deviations.

Tumor-specific cytotoxic T lymphocytes are considered to be a major component of the antitumor immune response. Treatment with CRAds may stimulate such a response through the induction of local inflammation at the site of tumor. Tumor specific CTL assays were performed by incubation of ^51^Cr-loaded autologous tumor cells with PBMC derived effector cells. Target cell lysis was determined by ^51^Cr release at effector cell to target ratios of 25:1, 50:1 and 100:1. Specific lysis was calculated as a percentage of released isotope compared to total releasable isotope available [18] (Figure 7). Lysis rates were low in all cultures, however some increase in CTL activity were detected in patients 2 and 3. No consistent pattern of CTL activity was evident. Cell lysis rates at all three ratios (1:25, 1:50, and 1:100) were not significant as determined by Paired t-test (1:25, Paired T-test; 0.38, P-value = .7130; 1:50, Paired T-test; -0.92, P-value = .3823; 1:100, Paired T-test; -0.85, P-value = .4175).

### 3.9 Enrollment and life span of patients

Eleven client-owned dogs with diagnoses of OSA (biopsy) were enrolled in the study (Table 1). Ten of those dogs successfully completed the protocol. All the dogs had been administered the canine hepatitis vaccine (CAV2) within the past 3 years. The affected leg was amputated in 10 dogs. One dog's (Dog 1) owners elected a limb-sparing surgery to remove the distal ulnar tumor, leaving the radius intact and the dog weight-bearing. Dog 7, 8, and 10 had suspected metastasis to lung or regional lymph nodes at the time of enrollment. Following 3 days recovery in the intensive care unit (ICU), a dose of 2 × 10^12^ viral particles of OC-CAVE1 CRAd was administered intravenously. The dogs were hospitalized, clinically monitored and regularly sampled for an additional 6 days. One dog (Patient 11) died approximately 12 h after receiving the virus. Post-mortem examination of this dog revealed signs of gas gangrene, including deep tissue necrosis and formation of gas pockets in the deep muscle tissues proximal to the amputation site. *Clostridium perfringens* was isolated from these tissues. The cause of death was judged to be due clostridial toxicosis secondary to surgery and the dog was excluded from all subsequent analyses. Vital signs, CBC, and serum chemistries for the 10 remaining dogs were within normal limits at every sampling time point. Following this period, the dogs were discharged to their owners and were asked to return after three weeks to draw a blood sample before starting on chemotherapy. Dogs were not monitored after their 4-week visit, for remainder of their survival.

**Figure 8:**
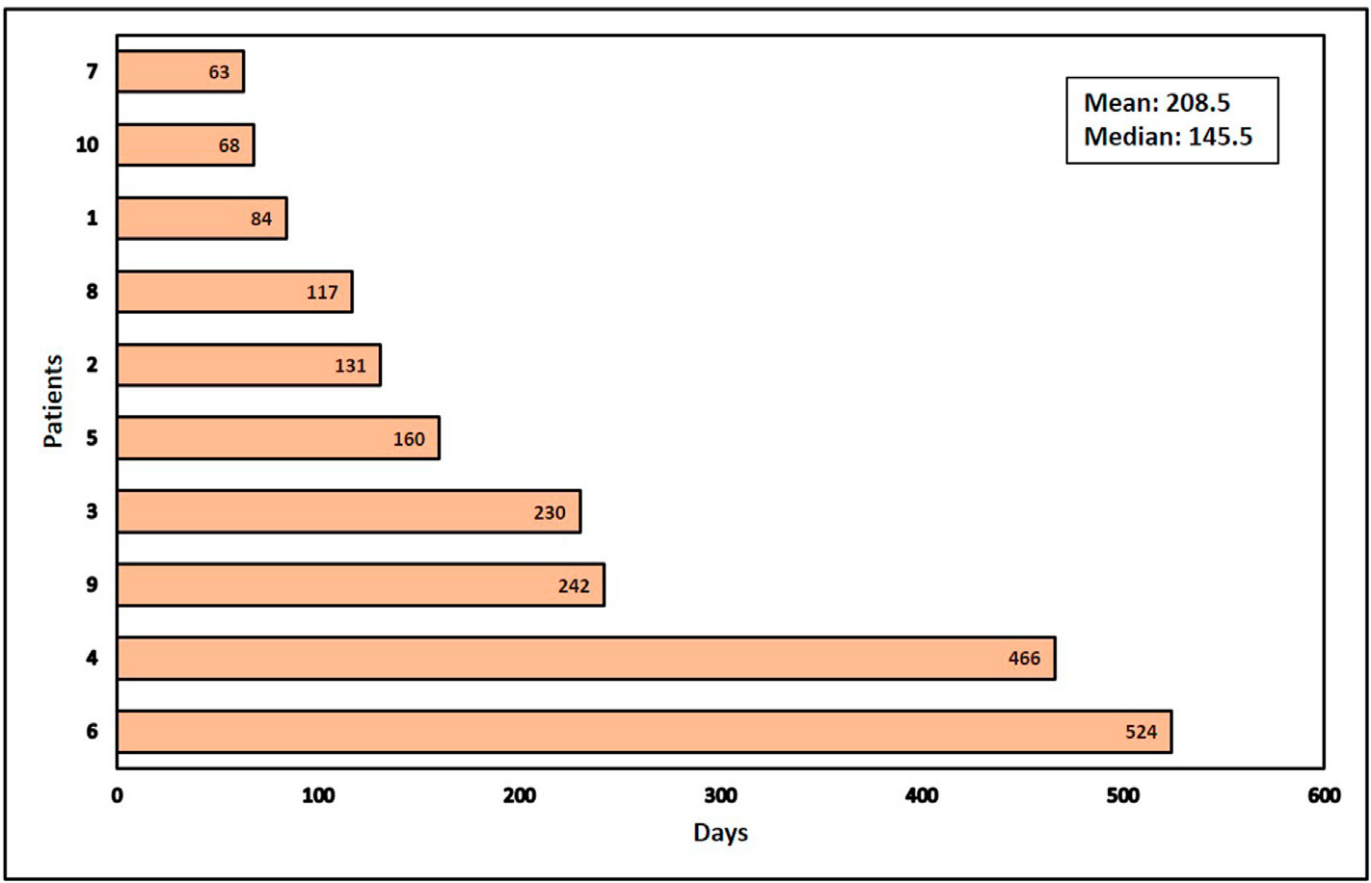
**S**urvival plot of study patients. The plot is graphed based on the days of survival post-virus injection. Mean survival time of all the dogs enrolled was 208.5 days and the medial survival was 145.5.

All 10 dogs that completed the study have now died, allowing complete mortality and longevity data to be collected (Table 1). Dogs 1, 5, 6, 7, 8, and 9 were confirmed to have died from OSA with dogs 2 and 10 confirmed to have died from other causes. The cause of death was not confirmed in dogs 3 and 4. Median survival time for the 10 dogs in the study with OSA was 145.5 days, while Mean survival was 208.5 days (Figure 8). Two of the ten dogs, dogs 4 and 6, survived well past one year, with survival times of 466 and 524 days (Figure 8). As noted above, the cause of death in dog 4 is unknown and in dog 6 was due to metastatic tumors (sarcoma) to the skin. This gives an overall 20% greater than one-year survival.

## 4 Discussion

OC-CAVE1 is a conditionally replicative canine adenovirus designed to replicate in cells that express the osteocalcin gene [22], such as OSA. This CRAd has been shown to effectively kill canine OSA cells in vitro and in a xenogeneic murine transplant model and has been shown to be safe in healthy dogs [15, 16]. CRAds such as OC-CAVE1 are hypothesized to work through several mechanisms. Viral replication leads to cell lysis and release of additional virus into the cell's microenvironment and the circulation. These new viral particles have the potential to infect new cells. Existing anti-viral immunity may respond to viral infection and replication in tumor cells with an anti-viral response, leading to additional tumor cell lysis and the local release of cytokines in the tumor microenvironment. The inflammatory response in turn, may shift the tumor microenvironment to one that supports the expansion of pre-existing anti-tumor immunity and the induction of novel immune responses against tumor antigens [23]. Thus, OC-CAVE1 administration may result in tumor cell killing by a combination of direct lysis, anti-viral immune responses, and anti-tumor immune responses. Ultimately, these mechanisms may be effective against both primary and metastatic disease.

Based on the potential for anti-tumor immune responses, we designed a pre-clinical study to evaluate the efficacy of OC-CAVE1 CRAd in inducing immune responses against potential OSA CSC and metastatic cells. We enrolled 10 patients in the study over a 2-year span. The dose of 2 × 10^12^ virus particles was selected based on the virus safety studies. That dose was derived by scaling the effective murine dose [16] to an approximate canine body mass. The rationale for the timing of administration following amputation included the concern that the primary tumor might serve as a “sink” for virus, removing it from the circulation and not allowing it to infect metastases and that significant killing of primary tumor could result in tumor lysis syndrome. Administration immediately following amputation was intended to address micrometastases that are assumed to be present in the vast majority of dogs, due to their subsequent development of gross metastatic disease. Ten dogs successfully completed the protocol and have now died, either from OSA or other causes.

Median survival time for dogs with OSA who receive amputation and aggressive chemotherapy is approximately 12 months, with 10–15% of dogs surviving long-term [8, 9, 10]. Median survival time in this study was 145.5 days and mean survival was 208.5 days, which are both considerably less than that. This figure may reflect the increased variation present due to the small number of animals enrolled. Two of the ten dogs, dogs 4 and 6, survived well past one year, with survival times of 466 and 524 days. Thus, the overall conclusion is that treatment with OC-CAVE1 did not statistically improve lifespan. However, there appears to a cohort of dogs in this study with relative longevity and the question must be asked whether that longevity is related to CRAd therapy, including CRAd induced immunity, or whether these dogs have endogenous mechanisms that allow them to more effectively fight this tumor. This study did not specify chemotherapeutic regimens to be employed following the study period so it is recognized that variability or inconsistency in those protocols may also have affected survival times.

Multiplication of virus particles in CSC and metastatic cells is expected in OSA patients. Quantitative PCR indicated that most patients had circulating viral particles detectable in blood for at least 6 days and the highest virus DNA amounts in blood at 12 h after virus injection. However, dogs 2, and 6 had clear viral peaks 48–60 h post infection, respectively. These peaks show that the virus underwent replication, which most likely occurred in micrometastases. Dog 6 had the most obvious peak of viral replication and was the longest survivor in the study. However, dog 2, who also showed apparent replication, reached neither the median nor mean survival. The second long-term survivor, dog 4, did not show an obvious peak of virus, but did have one of the highest levels of circulating virus over the course of the study.

Virus DNA was identified sporadically in urine and feces. Fecal or urinary shedding of viral DNA was not evident in all patients and the presence of shed DNA did not appear to correlate with any other phenomenon connected with the study. If shed viral DNA represented infectious viral particles, this could represent an infection hazard for other animals that were exposed to the urine and feces of treated dogs for a period in excess of a week following injection. However, multiple attempts to culture viable viral particles from feces or urine failed to produce any evidence of viable virus. Human CRAd patients have also not been documented to transmit CRAds to other people [24].

In response to external antigens, the body's immune system generates targeted antibodies against the antigen. We hypothesized that in response to our vector, there would be an enhanced cellular and humoral immune response against CAV2 and tumor neo-antigens due to enhanced exposure through cell lysis and the release of cytokines from immune effector cells upon contact with virus or virus infected cells. Plasma was evaluated for CAV2 antibody titers. Increases of 5- to 125- fold in reciprocal CAV2 antibody titers in plasma 4 weeks after OC-CAVE1 injection indicates a strong anamnestic humoral immune response against CAV2.

IgG responses to autologous tumor cells were assessed by Western blot and flow cytometry. Plasma from patient dogs was used as a primary polyclonal antibody to perform Western blot analysis with autologous tumor cell lysates. These blots show multiple bands of interest in both the pre and 4 weeks post treatment samples. A few of these bands were of higher intensity when probed with post-virus injection plasma when compared to pre-injection plasma, indicating that humoral immune responses to some pre-existing antigens were enhanced in some dogs.

Flow cytometry indicated no real difference between the number of tumor cells recognized by the IgG response against tumor antigens serum before and after treatment in every dog except dog 1. Dog 1 showed an increase of nearly 25% in the percentage of cells staining with autologous serum. Interestingly, dog 1 is the only dog on the study that underwent limb-sparing surgery and its tumor recurred at the primary site at around the time that the second sample (4 weeks after virotherapy) was taken. The dog was euthanized shortly thereafter for progressive disease and discomfort. Thus, the increase in the number of cells bound by antibody in this dog may reflect a higher antigen load.

These results indicate that OC-CAVE1 CRAd did result both in an increase in pre-existing anti-tumor humoral responses and the production of antibodies to additional proteins post administration in some patients. However, the percentage of tumor cells recognized did not increase, nor did there appear to be any survival benefit to any of the observed humoral responses.

We have also evaluated the levels of dendritic and regulatory T cells in peripheral blood prior to and following administration of the virus, using flow cytometry and chromium release assays to monitor cell-mediated immune responses.

CD11c is a transmembrane protein expressed on the cell surface of phagocytic immune cells such as dendritic cells, monocytes, macrophages, and neutrophils. CD11c is the hallmark surface marker of dendritic cells and is used as a dendritic cell specific cell surface marker along with MHC class II molecules for flow cytometry quantification [25]. In this study we quantified the CD11c + population in order to monitor the effect of CRAd treatment on antigen presenting cells. Flow cytometry results indicated an increase in CD11c + populations in dogs 6 and 10. Given that dog 6 was a long-term survivor, that might imply a correlation, however, dog 10 was one of the shortest-term survivors, making any impact of increased dendritic cells difficult to interpret.

Regulatory T cells are a differentiated subset of CD4+ T cells that have an active role of immune suppression against self-antigens. Differentiated, functional T-reg cells have cell surface receptors CD4 and FoxP3 among others and are associated with suppressed anti-tumor immunity. Our results indicate that T-reg cell population was significantly decreased in treated patients. These results indicate that T-reg populations in the circulation may be reduced in response to CRAd treatment, although the decrease may also be related to the removal of the primary tumor as a source of PD-L1 or other checkpoint inhibitors [26]. Regardless of the cause, the decrease in circulating T-regs is promising in that it may indicate that the immune system in these patients is entering a state where a functional anti-tumor immune response is possible.

It is important to note that both dendritic and regulatory T-cells are non-specific measures of immune response. Given the circumstances in which these dogs were sampled, immediately following amputation and 4 weeks after administration of the CRAd, there are many additional factors that could influence, or mask, these responses, including surgery, wound healing, and administration of anti-inflammatory medicines. Additionally, time and sampling constraints limited the sampling times. Ideally, future studies should obtain samples prior to surgery, post-surgery and weekly to assess these and other non-specific immune parameters.

Cytotoxic T-cell assays were performed using autologous tumor cells as targets. These assays indicated the ability of the patient's T-cells to kill the patient's tumor before and after treatment. Unfortunately, a consistent pattern in CTL activity was not observed in any of the patients except for an increase in CTL activity in patients 2 and 3. While this increase in activity is encouraging, it is not correlated with survival.

Dogs 7, 8, and 10 had the appearance of pre-existing metastatic tumor on chest radiographs, but none of the results of these dogs differed from dogs with no obvious metastatic growth.

## 5 Conclusion

Our study was designed with the hypothesis that intravenous administration of a CAV2 based CRAd, in dogs that had been amputated for primary OSA, would infect micrometastatic cells and cause cell lysis. This cell lysis and exposure of viral vector and tumor antigens was hypothesized to induce humoral and cell mediated immune responses to tumor in patients. While some encouraging changes in immunological parameters were observed in this study, there was no measurable impact on survival in the cohort studied. The time of administration of the CRAd was designed to address micrometastases as rapidly as possible after diagnosis to allow a 4-week period to study the immune response before administering potentially immunosuppressive chemotherapy. In reality, it might be better to treat with chemotherapy first and reserve virotherapy for a later time when metastatic disease is clearly present. However, there was no significant effect either in dogs [7, 8, 10] with suspected metastatic growth.

There are multiple other possible explanations for this outcome, including the small number of animals, relatively slow replication characteristics of this particular CRAd, low levels of activity from the osteocalcin promoter in tumor, too few tumor cells to support replication in most dogs due to removal of the primary lesion, and a failure to overcome immune checkpoint inhibitors expressed by tumors, such as PD-L1.

## Declarations

### Author contribution statement

Payal Agarwal: Conceived and designed the experiments; Performed the experiments; Analyzed and interpreted the data; Contributed reagents, materials, analysis tools or data; Wrote the paper.

Elizabeth A Gammon: Performed the experiments.

Maninder Sandey, Jey W Koehler: Analyzed and interpreted the data.

Stephanie S Lindley, Brad M Matz, Annette N Smith, Elena A. Kashentseva, Igor P. Dmitriev, David T Curiel: Contributed reagents, materials, analysis tools or data.

Bruce F Smith: Conceived and designed the experiments; Analyzed and interpreted the data; Contributed reagents, materials, analysis tools or data.

### Funding statement

This work was supported by American Kennel Club Canine Health Foundation (01806).

### Data availability statement

Data included in article/supplementary material/referenced in article.

### Declaration of interests statement

The authors declare no conflict of interest.

### Additional information

No additional information is available for this paper.

## References

[1] Endicott M. (2003). Principles of treatment for osteosarcoma. Clin. Tech. Small Anim. Pract..

[2] Dernell W.S., Vail D. (2007). Tumors of the skeletal system. MacEwans's Small Animal Clinical Oncology.

[3] Rosenberger J.A., Pablo N.V., Crawford P.C. (2007). Prevalence of and intrinsic risk factors for appendicular osteosarcoma in dogs: 179 cases (1996-2005). J. Am. Vet. Med. Assoc..

[4] Morello E., Martano M., Buracco P. (2011). Biology, diagnosis and treatment of canine appendicular osteosarcoma: similarities and differences with human osteosarcoma. Vet. J. (Lond, Engl.: 1997).

[5] Phillips B., Powers B.E., Dernell W.S., Straw R.C., Khanna C., Hogge G.S. (2009). Use of single-agent carboplatin as adjuvant or neoadjuvant therapy in conjunction with amputation for appendicular osteosarcoma in dogs. J. Am. Anim. Hosp. Assoc..

[6] Pesonen S., Kangasniemi L., Hemminki A. (2011). Oncolytic adenoviruses for the treatment of human cancer: focus on translational and clinical data. Mol. Pharm..

[7] Short J.J., Curiel D.T. (2009). Oncolytic adenoviruses targeted to cancer stem cells. Mol. Canc. Therapeut..

[8] Gibbs C.P., Levings P.P., Ghivizzani S.C. (2011). Evidence for the osteosarcoma stem cell. Curr. Orthop. Pract..

[9] Siclari V.A., Qin L. (2010). Targeting the osteosarcoma cancer stem cell. J. Orthop. Surg. Res..

[10] Bourke M.G., Salwa S., Harrington K.J., Kucharczyk M.J., Forde P.F., de Kruijf M. (2011). The emerging role of viruses in the treatment of solid tumours. Canc. Treat Rev..

[11] Mason N.J., Gnanandarajah J.S., Engiles J.B., Gray F., Laughlin D., Gaurnier-Hausser A. (2016). Immunotherapy with a HER2-targeting Listeria induces HER2-specific immunity and demonstrates potential therapeutic effects in a Phase I trial in canine osteosarcoma. Clin. Cancer. Res. – Offic. J. Am. Assoc. Cancer Res..

[12] Prestwich R.J., Errington F., Diaz R.M., Pandha H.S., Harrington K.J., Melcher A.A. (2009). The case of oncolytic viruses versus the immune system: waiting on the judgment of Solomon. Hum. Gene Ther..

[13] Crittenden M.R., Thanarajasingam U., Vile R.G., Gough M.J. (2005). Intratumoral immunotherapy: using the tumour against itself. Immunology.

[14] Biller B.J., Guth A., Burton J.H., Dow S.W. (2010). Decreased ratio of CD8+ T cells to regulatory T cells associated with decreased survival in dogs with osteosarcoma. J. Vet. Intern. Med./Am. College Vet. Intern. Med..

[15] Smith B.F., Curiel D.T., Ternovoi V.V., Borovjagin A.V., Baker H.J., Cox N. (2006). Administration of a conditionally replicative oncolytic canine adenovirus in normal dogs. Cancer Biother. Radiopharm..

[16] Hemminki A., Kanerva A., Kremer E.J., Bauerschmitz G.J., Smith B.F., Liu B. (2003). A canine conditionally replicating adenovirus for evaluating oncolytic virotherapy in a syngeneic animal model. Mol. Ther. – J. Am. Soc. Gene Ther..

[17] (2016). Veterinary cooperative oncology group - common terminology criteria for adverse events (VCOG-CTCAE) following chemotherapy or biological antineoplastic therapy in dogs and cats v1.1. Vet. Comp. Oncol..

[18] Bird R.C., Deinnocentes P., Lenz S., Thacker E.E., Curiel D.T., Smith B.F. (2008). An allogeneic hybrid-cell fusion vaccine against canine mammary cancer. Vet. Immunol. Immunopathol..

[19] Helfand S.C., Soergel S.A., Modiano J.F., Hank J.A., Sondel P.M. (1994). Induction of lymphokine-activated killer (LAK) activity in canine lymphocytes with low dose human recombinant interleukin-2 in vitro. Cancer Biother..

[20] Helfand S.C., Dickerson E.B., Munson K.L., Padilla M.L. (1999). GD3 ganglioside antibody augments tumoricidal capacity of canine blood mononuclear cells by induction of interleukin 12. Cancer Res..

[21] Khanna C., Hasz D.E., Klausner J.S., Anderson P.M. (1996). Aerosol delivery of interleukin 2 liposomes is nontoxic and biologically effective: canine studies. Clin. Cancer. Res. – Offic. J. Am. Assoc. Cancer Res..

[22] Wehrle-Martinez A.S., Dittmer K.E., Aberdein D., Thompson K.G. (2016). Osteocalcin and osteonectin expression in canine osteosarcoma. Veterinary pathology.

[23] Ricca J.M., Oseledchyk A., Walther T., Liu C., Mangarin L., Merghoub T. (2018). Pre-existing immunity to oncolytic virus potentiates its immunotherapeutic efficacy. Mol. Ther. – J. Am. Soc. Ther..

[24] Wold W.S., Toth K. (2013). Adenovirus vectors for gene therapy, vaccination and cancer gene therapy. Curr. Gene Ther..

[25] Poltorak M.P., Schraml B.U. (2015). Fate mapping of dendritic cells. Front. Immunol..

[26] Zuazo M., Gato-Canas M., Llorente N., Ibanez-Vea M., Arasanz H., Kochan G. (2017). Molecular mechanisms of programmed cell death-1 dependent T cell suppression: relevance for immunotherapy Pre-existing Immunity to Oncolytic Virus Potentiates its Immunotherapeutic Efficacy. Ann. Transl. Med..

