# Supplementary material for: Evaluation of tumor immunity after administration of conditionally replicative adenoviral vector in canine osteosarcoma patients
Source: Heliyon. 2021 Feb 10;7(2):e06210. doi: 10.1016/j.heliyon.2021.e06210 (PMC7881234; doi:10.1016/j.heliyon.2021.e06210)

Data S1: Western blots from each patient as cropped and uncropped images, Related to Figure 3

A) Western Blots: Cropped and adjusted for dimensions

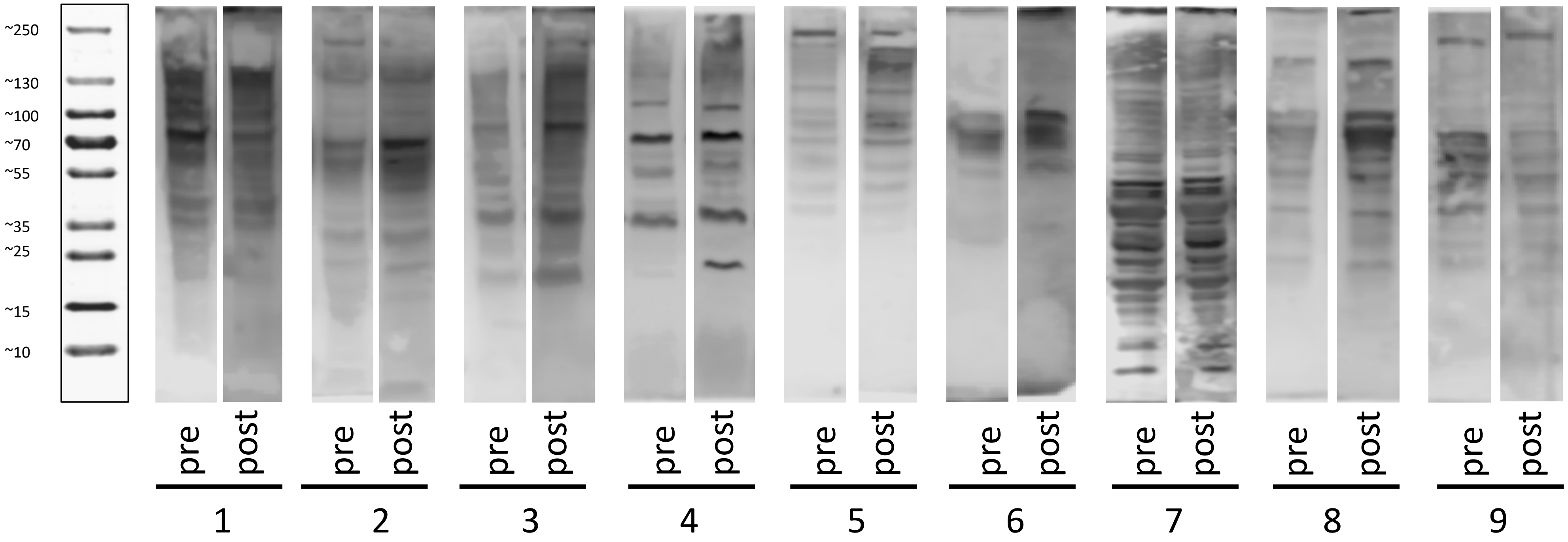

B) Western Blots Uncropped and unadjusted

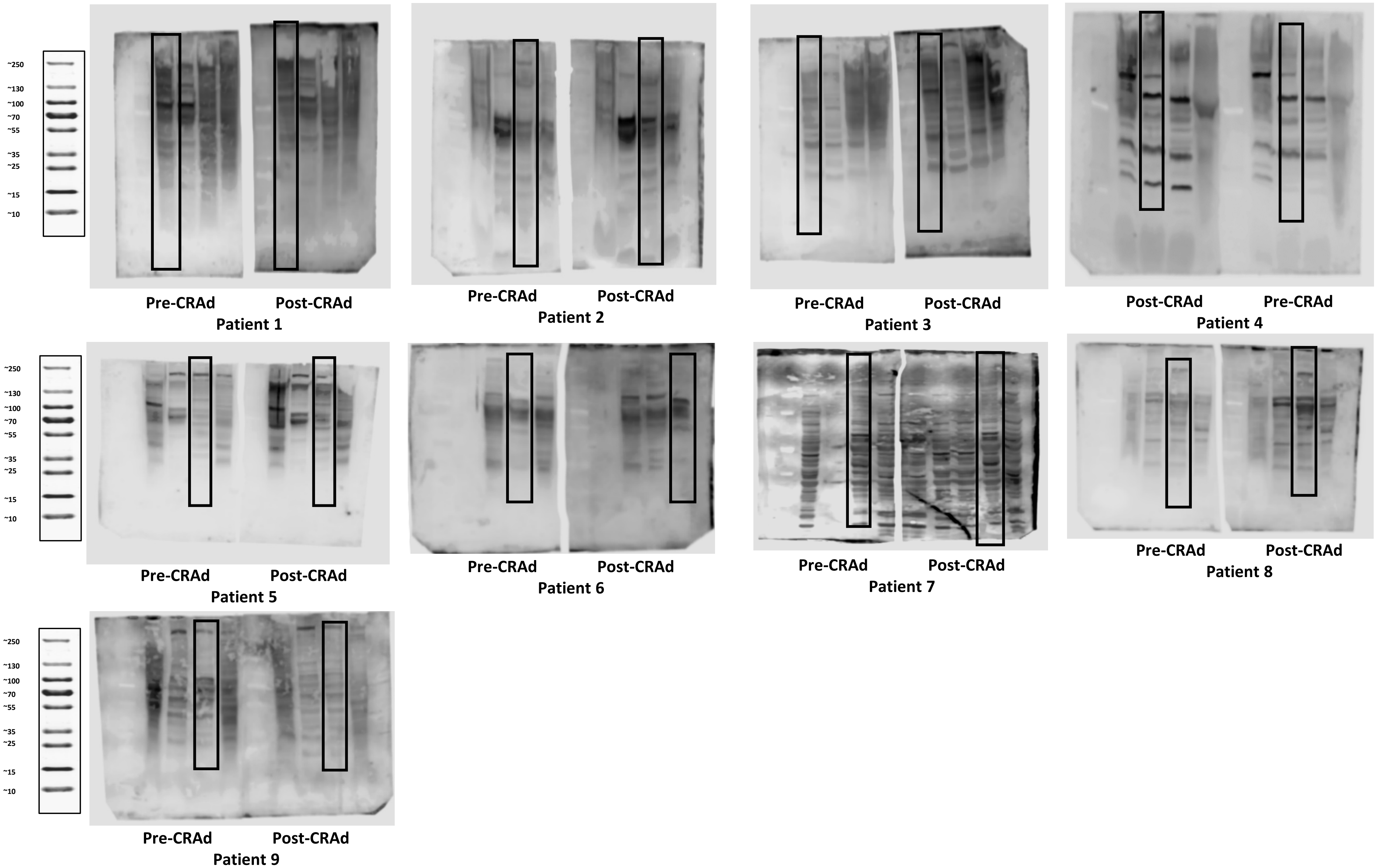

Supplement: Supplemental Material.pdf [file mmc1.pdf]
